# microRNA-22, downregulated in hepatocellular carcinoma and correlated with prognosis, suppresses cell proliferation and tumourigenicity

**DOI:** 10.1038/sj.bjc.6605895

**Published:** 2010-09-14

**Authors:** J Zhang, Y Yang, T Yang, Y Liu, A Li, S Fu, M Wu, Z Pan, W Zhou

**Affiliations:** 1 The Third Department of Hepatic Surgery, Eastern Hepatobiliary Surgery Hospital, Second Military Medical University, Shanghai, China; 2 Department of Pharmacy, Shanghai General Hospital of Chinese People's Armed Police Forces, Shanghai, China

**Keywords:** miR-22, hepatocellular carcinoma, prognosis, proliferation, HDAC4

## Abstract

**Background:**

Recently, microRNAs in cancer development have attracted much attention, but their roles in tumorigenesis are still largely unknown. In this study, a functional role of miR-22 in hepatocellular carcinoma (HCC) development has been identified.

**Methods:**

Quantitative real-time PCR was used to determine the level of miR-22 transcript in HCC clinical samples, and its correlation with disease-free survival was determined using Kaplan–Meier method. Restoration of miR-22 expression was carried out in HCC cell lines to assess its influence on HCC cell proliferation and tumourigenicity.

**Results:**

In the 160 paired HCC tissue samples, miR-22 expression was downregulated in HCC, and low miR-22 expression in HCC was predictive of poor survival in HCC patients. Functional studies indicated that ectopic expression of miR-22 significantly inhibits HCC cell proliferation and tumourigenicity. Furthermore, histone deacetylase 4 (HDAC4), known to have critical roles in cancer development, was proved to be directly targeted and regulated by miR-22. Furthermore, HDAC4 was upregulated in miR-22-downregulated HCC tissues, suggesting that downregulation of miR-22 might participate in HCC carcinogenesis and progression through potentiation of HDAC4 expression. In addition, cell proliferation was also suppressed by knockdown of HDAC4 or treatment with HDAC inhibitor trichostatin A in HCC cell lines.

**Conclusion:**

miR-22, downregulated in HCC, has an anti-proliferative effect on HCC cells both *in vitro* and *in vivo*. Furthermore, miR-22 may have considerable potential in identification of the prognosis and application of cancer therapy for HCC patients.

MicroRNAs (miRNAs) are an abundant class of 17–25 nucleotides small noncoding RNAs. They regulate protein-coding genes expression at the post-transcriptional level through binding to the 3′ untranslational region (3′ UTR) of target mRNAs. Since the initial observation, ∼1000 miRNA sequences have been identified in mammals, but the biological functions of a large part remain illusive. Growing evidence have suggested that miRNAs have important roles in the regulation of diverse biological processes (Bushati and Cohen, 2007), and their deregulation or dysfunction participates in cancer development and clinical outcomes of cancer patients (Garzon *et al*, 2009). However, deregulated miRNAs and their roles in tumorigenesis are still largely unknown.

Hepatocellular carcinoma (HCC) is the fifth most common cancer worldwide and among the leading causes of cancer-related deaths (Lai and Lau, 2005). To date, miRNAs have been suggested to have important roles in HCC development (Gramantieri *et al*, 2008), and some of them have been identified to correlate with prognosis or accepted to be potential therapeutic targets (Li *et al*, 2008; Ji *et al*, 2009; Xiong *et al*, 2009). However, elucidating miRNA deregulation or dysfunction in HCC development is still an ongoing process. Recently, miR-22 has been shown to be deregulated in some other types of cancers, such as overexpression in prostate cancer and downregulation in breast cancer, cholangiocarcinoma, and multiple myeloma (Kawahigashi *et al*, 2009; Lionetti *et al*, 2009; Poliseno *et al*, 2010; Xiong *et al*, 2010). However, the roles of miR-22 deregulation in carcinogenesis and progression remain largely elusive. Hence, in this study, we focus on the expression and function of miR-22 in HCC development.

In this study, in the 160 HCC and matched non-neoplastic tissues, we found that miR-22 expression was downregulated, and its downregulation correlated with decreased disease-free survival of HCC patients. In HCC cell lines, we further investigated the roles of miR-22 in cell proliferation both *in vitro* and *in vivo*, and found that miR-22 may function as a tumour suppressor. In addition, histone deacetylase 4 (HDAC4), known to have critical roles in cancer development, is proved to be targeted and regulated by miR-22, indicating that downregulation of miR-22 may involve in HCC carcinogenesis and progression through potentiation of HDAC4 expression. Thus, we elucidated the roles of miR-22 downregulation in HCC development and suggested a potential target in identification of the prognosis and application of cancer therapy for HCC patients.

## Materials and methods

### Patients and tissue samples

Surgically resected paired HCC and adjacent non-neoplastic tissues were collected from 160 primary HCC patients during operation in 2006 at the Eastern Hepatobiliary Surgery Hospital, and the details are shown in (Supplementary Table 1). Human normal liver tissue samples were obtained during operation from 10 normal liver tissue of liver hemangioma patients without hepatitis virus infection. Surgically removed tissues were quickly frozen in liquid nitrogen until analysis. All samples were collected with the informed consent of the patients and the experiments were approved by the ethics committee of Second Military Medical University, Shanghai, China. The investigations were conducted according to the Declaration of Helsinki Principles.

### RNA extraction and quantitative RT-PCR

Total RNA, including miRNA, was extracted using TRIzol reagent (Invitrogen, Carlsbad, CA, USA) according to the manufacturer’s instructions. miRNA detection was performed using TaqMan miRNA assay system (Applied Biosystems, Foster City, CA, USA) as described by us previously (Chen *et al*, 2009). The expression of miR-22 was normalised to that of internal control 18S rRNA by using 2^−ΔΔ*C*_t_^ (cycle threshold) method (Hou *et al*, 2009; Livak and Schmittgen, 2001). For miR-22 copy number estimation, we followed previously reported protocols of detecting miRNA copy numbers (Chang *et al*, 2004; Sarasin-Filipowicz *et al*, 2009). In brief, we generated standard curves using HPLC- and PAGE-purified oligoribonucleotides (GenePharma, Shanghai, China) corresponding to miR-22. The cycle threshold values obtained from real-time quantitative PCR reactions were converted to absolute copy number of miR-22 per 20 pg of total RNA from liver tissue, using miR-22 standardisation curves.

### Cell culture and transfection

Human HCC cell lines Hep3B and SMMC7721 were obtained from American Type Culture Collection (Manassas, VA, USA) and cultured in DMEM (Invitrogen) supplemented with heat-inactivated 10% fetal bovine serum (Invitrogen) at 37°C in a humidified incubator containing 5% CO_2_. For RNA transfection, 5 × 10^3^ Hep3B or SMMC7721 cells were seeded into each well of 96-well plate and incubated overnight, then transfected with RNAs using INTERFERin transfection reagent (Polyplus transfection, Illkirch, France) at a final concentration of 50 nM according to the manufacturer’s instructions.

### miRNA and siRNA

For the gain-of-function study, miR-22 mimics (double-stranded RNA oligonucleotides) and scrambled control RNA were synthesised by GenePharma. The scrambled control RNA sequences were 5′-UUCUCCGAACGUGUCACGUTT-3′ (sense) and 5′-ACGUGACACGUUCGGAGAATT-3′ (anti-sense). For human HDAC4 knockdown, specific small-interfering RNA (siRNA) and scrambled control RNA were purchased from Santa Cruz Biotechnology (Santa Cruz, CA, USA).

### Analysis of cell proliferation *in vitro*

The *in vitro* cell proliferation of HCC cell lines was measured using the MTT method (Alley *et al*, 1988). In brief, cells were seeded into 96-well plates and transfected. In the indicated time periods, 0.1 ml of spent medium was replaced with an equal volume of fresh medium containing MTT 0.5 mg ml^−1^. Plates were incubated at 37°C for 4 h, and then the medium was replaced by 0.1 ml of DMSO (Sigma, St Louis, MO, USA) and plates were shaken at room temperature for 10 min. The absorbance was measured at 570 nm.

### Tumourigenicity assay in nude mice

All experiments involving animals were undertaken in accordance with the National Institute of Health *Guide for the Care and Use of Laboratory Animals*, with the approval of the Scientific Investigation Board of Second Military Medical University, Shanghai. The tumourigenicity assay was performed as reported (Su *et al*, 2009; Xiong *et al*, 2009). In detail, 5 × 10^6^ control RNA or miR-22 mimics transfected HCC Hep3B or SMMC7721 cells were suspended in 0.1 ml PBS and then injected subcutaneously into either side of the posterior flank of the same male BALB/c athymic nude mice at 4 weeks of age. Tumour growth was measured using caliper daily, and tumour volume was calculated according to the formula: volume=length × width^2^ × 0.5.

### 3′ UTR luciferase reporter assay

The human HDAC4 3′ UTR luciferase reporter construct was generated by cloning HDAC4 mRNA 3′ UTR sequence into the 3′ UTR region of pMIR-Report construct (Ambion, Foster City, CA, USA). The miR-22 target site-deleted HDAC4 3′ UTR luciferase reporter construct was generated by PCR fragments of HDAC4 3′ UTR luciferase reporter construct lacking the target site and ligated. Hep3B cells were transfected and luciferase activities were measured as described by us previously (Chen *et al*, 2009). Data were normalised by dividing Firefly luciferase activity with that of *Renilla* luciferase.

### Western blot

Cells and ground tissues were lysed, equalised, loaded, and blotted as we described previously (Chen *et al*, 2009). Antibodies specific to HDAC4, *β*-actin, and horseradish-peroxidase-coupled secondary antibodies were purchased from Santa Cruz Biotechnology. Densitometric analysis was carried out with Labworks Image Acquisition and Analysis software (UVP, Upland, CA, USA). The background was subtracted, and the signals of the detected bands were normalised to the amount of loading control *β*-actin band. The relative value was presented as fold increase over control sample as indicated.

### Statistical analysis

Data are presented as mean±s.d. Statistical comparisons between experimental groups were analysed by Student's *t*-test and a two-tailed *P*<0.05 was taken to indicate statistical significance. For analysing survival of HCC patients, log-rank test in SPSS 17.0 (Chicago, IL, USA) was used with the *P*-value indicated.

## Results

### miR-22 is downregulated in HCC

**Figure 1:**
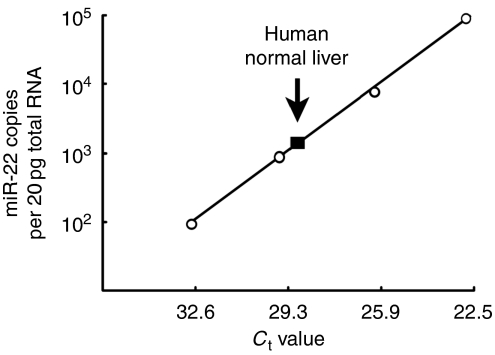
Endogenous miR-22 expression level in human normal liver. Data are shown as miRNA copies per 20 pg total RNA (equivalent of approximately one cell).

To investigate the roles of miR-22 in HCC development, we determined the expression level of miR-22 in human normal liver, as it was reported that a minimum threshold amounts must be reached for miRNAs to repress their target mRNAs (Brown *et al*, 2007; Sarasin-Filipowicz *et al*, 2009). In human distal normal liver tissue of liver hemangioma obtained from 10 patients during operation, we found that miR-22 was expressed at about 2 × 10^3^ copies per 20 pg total RNA (Figure 1), equivalent of approximately one cell. Thus, the expression of miR-22 in human normal liver is much higher than the required amount of miRNA for targets repression, which is ∼100 copies per cell (Brown *et al*, 2007; Sarasin-Filipowicz *et al*, 2009).

**Figure 2:**
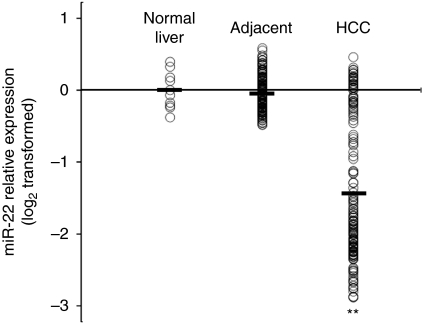
miR-22 expression is downregulated in HCC. Data are shown as miR-22 expression in 10 human normal liver samples, and 160 paired HCC and adjacent non-neoplastic tissue samples, with the black line showing the medium value. ^**^P<0.01.

We further investigated the expression of miR-22 in 160 HCC and matched adjacent non-neoplastic tissue samples. As shown in Figure 2, miR-22 expression is significantly decreased in at least 69% (111 of 160) HCC samples compared with their matched controls. Hence, we for the first time show that miR-22 is downregulated in HCC, which may contribute to HCC pathogenesis.

### Downregulation of miR-22 correlates with worse prognosis of HCC patients

**Figure 3:**
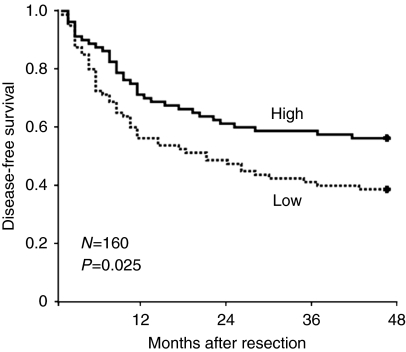
Kaplan–Meier disease-free survival analysis for HCC patients according to miR-22 level. The median value of miR-22 level was chosen as the cutoff point for separating miR-22 low-expression tumours (*n*=80) from miR-22 high-expression cases (*n*=80). *P*-value is shown with the use of log-rank test in SPSS 17.0.

Whether miR-22 downregulation correlates with prognosis of HCC patients was investigated further. As shown in Figure 3, the Kaplan–Meier method reveals that lower miR-22 expression level correlates with significantly reduced disease-free survival of the 160 HCC patients. This result further indicates the importance of miR-22 downregulation in HCC development.

### miR-22 suppresses cell proliferation and tumourigenicity of HCC cell lines

**Figure 4:**
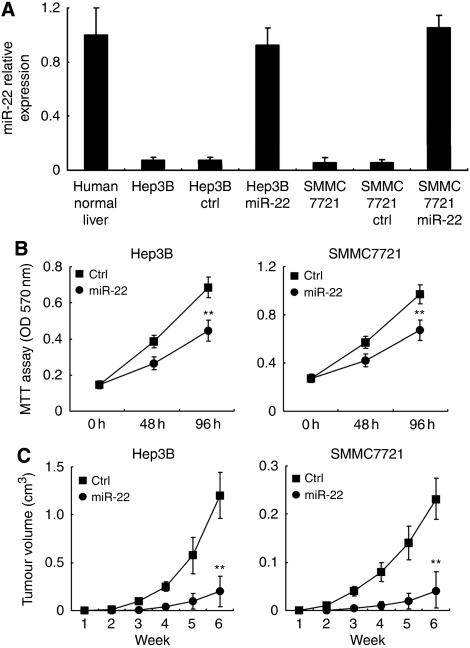
miR-22 suppresses cell proliferation and tumourigenicity. (**A**) miR-22 expression in HCC cell lines Hep3B and SMMC7721, and transfected with control (ctrl) RNA or miR-22 mimics for 4 days. (**B**) Cell proliferation of these cells transfected as in **A** was measured in the indicated time periods using MTT assay. (**C**) Effect of miR-22 on tumourigenicity in nude mouse xenograft model. The transfected Hep3B and SMMC7721 cells were injected subcutaneously into either side of the posterior flank of the same nude mice respectively. Tumour growth curve is shown as indicated. Data are shown as mean±s.d. (*n*=6). ^**^P<0.01.

The downregulated miR-22 in HCC prompted us to investigate whether miR-22 functions as a tumour suppressor. In HCC cell lines Hep3B and SMMC7721, we found that miR-22 expression was also markedly decreased (Figure 4A), and restoration of miR-22 expression suppressed cell proliferation in both of the HCC cell lines (Figure 4A and B). Furthermore, miR-22-transfected HCC Hep3B and SMMC7721 cells revealed a delayed tumour formation and a significant reduction in tumour size compared with that of control transfectants (Figure 4C). These results show that miR-22 restoration suppresses HCC growth both *in vitro* and *in vivo*.

### miR-22 targets HDAC4

**Figure 5:**
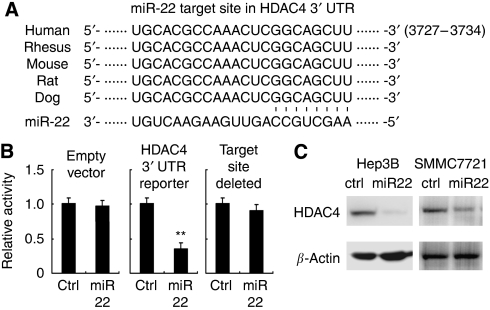
miR-22 targets human HDAC4. (**A**) Sequence alignment of miR-22 and its conserved target site in HDAC4 3′ UTR (downloaded from TargetScan). (**B**) Luciferase expression of the indicated construct co-transfected with control RNA or miR-22 mimics. Data are shown as mean±s.d. (*n*=6). ^**^P<0.01. (**C**) Hep3B and SMMC7721 cells were transfected as in Figure 4A. After 48 h, human HDAC4 and *β*-actin were detected by western blot.

Next, we sought to investigate the molecular mechanism responsible for the anti-tumour effect of miR-22 on HCC observed above. As miRNAs function mainly through inhibiting their target mRNAs by binding to the 3′ UTR, we searched the putative target genes of miR-22 in TargetScan (http://www.targetscan.org/). In the 330 predicted conserved targets, we found that human HDAC4, known to have critical roles in cancer development by repressing differentiation-promoting genes (Wade, 2001), contained putative conserved miR-22 target site (Figure 5A). To verify whether HDAC4 is a direct target of miR-22, a dual-luciferase reporter system was used by co-transfection of miR-22 and a luciferase reporter plasmid containing the 3′ UTR of human HDAC4. As shown in Figure 5B, the luciferase activity was significantly inhibited by miR-22 co-transfection, and further miR-22 failed to inhibit the expression of luciferase construct on target site deletion, suggesting that miR-22 can directly target the 3′ UTR of HDAC4. Moreover, in HCC Hep3B and SMMC7721 cells, endogenous expression of HDAC4 protein was suppressed by miR-22 transfection (Figure 5C), further proving that HDAC4 is directly targeted and regulated by miR-22 expression.

### HDAC4 is upregulated in miR-22-downregulated HCC tissues

**Figure 6:**
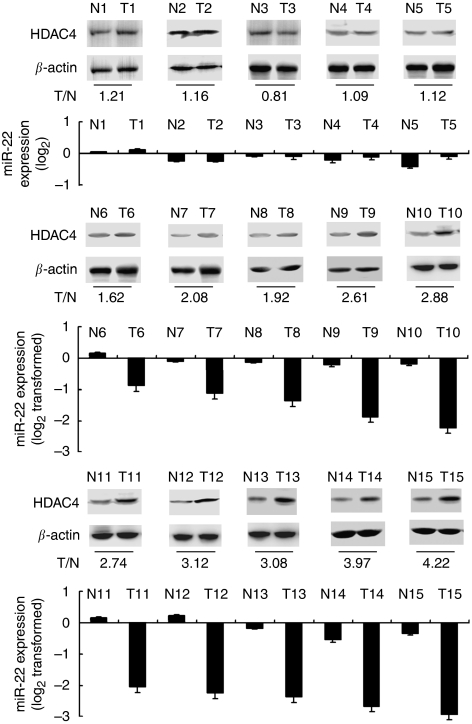
HDAC4 is upregulated in miR-22-downregulated HCC tissues. Expression of HDAC4 and *β*-actin in available protein samples of matched adjacent non-neoplastic liver tissues *vs* HCC tissues were detected by western blot, and miR-22 expression in these samples were shown below.

We further compared HDAC4 protein expression between primary HCC and matched non-neoplastic liver tissue samples, as miR-22 was observed to be downregulated in a large part of HCC tissues above. In available protein samples of paired HCC tissues, we found that HDAC4 expression was upregulated in miR-22-downregulated HCC tissues (Figure 6). This result further confirms that endogenous HDAC4 is regulated by miR-22 and miR-22 downregulation may participate in HCC carcinogenesis and progression through potentiation of HDAC4 expression.

### Inhibition of HDAC4 suppresses cell proliferation of HCC cell lines

**Figure 7:**
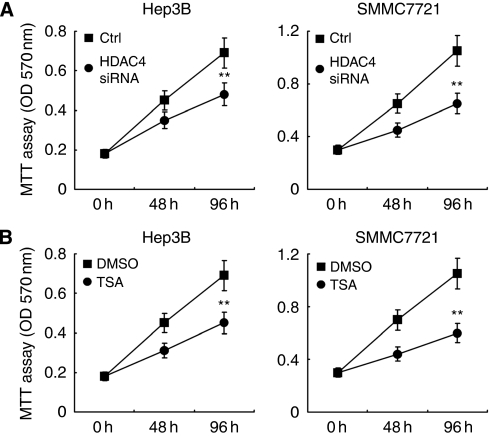
Inhibition of HDAC4 suppresses cell proliferation. Hep3B and SMMC7721 cells were transfected with control RNA or HDAC4 siRNA (**A**), or treated with TSA (100 nM) (**B**) for the indicated time periods. Cell proliferation was measured using MTT assay. Data are shown as mean±s.d. (*n*=6). ^**^P<0.01.

Next, we evaluated whether HCC cell growth is regulated by HDAC4 expression, which is targeted by miR-22. As shown in Figure 7, knockdown of HDAC4 or treatment with HDAC inhibitor trichostatin A (TSA) suppressed cell proliferation in HCC Hep3B and SMMC7721 cells. This result further suggests that miR-22 may suppress HCC cell proliferation at least partially through targeting HDAC4 expression.

## Discussion

In this study, we presented the downregulation of miR-22 in HCC and suggested the anti-tumour effect of miR-22 in HCC pathogenesis. Previous reports suggested that miR-22 is also downregulated in breast cancer, and it suppresses breast cancer development through directly targeting oestrogen receptor *α* (ER*α*) and downstream signalling (Pandey and Picard, 2009; Xiong *et al*, 2010). However, miR-22 expression is suggested to be upregulated in prostate cancer, and its upregulation potentiates phosphatidylinositol 3-kinase-Akt pathway activation (Poliseno *et al*, 2010). These controversial results of miR-22 in cancer development may reflect the diverse roles of miR-22 in different types of cancer.

Downregulation of miR-22 was suggested to exist in a large part of HCC tissues. However, underlying mechanisms that mediate the downregulation of miR-22 in HCC are still elusive. Epigenetic alterations, such as DNA methylation or histone modification, in cancer often mediate the deregulation of tumour suppressors or oncogenes (Esteller, 2008), and HDAC4 expression potentiated by miR-22 downregulation may further aggravate the epigenetic changes in HCC. We will next focus on the mechanisms that mediate miR-22 downregulation and miR-22-downregulation-induced epigenetic alterations in HCC pathogenesis.

Downregulation of miR-22 is further suggested to correlate with reduced disease-free survival of HCC patients. Hence, as HCC patients with normal miR-22 expression in tumour tissue are shown to have a better prognosis, determination of miR-22 expression level in HCC tissues may be a novel approach to predict and identify the prognosis of HCC patients.

The anti-tumour effect of miR-22 in HCC is validated both *in vitro* and *in vivo*, which is that restoration of miR-22 expression significantly suppresses cell proliferation and tumourigenicity. Recently, reexpression of miRNAs is believed to hold substantial clinical potential in cancer therapy (Mishra and Merlino, 2009). As systemic chemotherapy for HCC has been quite ineffective (Liu *et al*, 2006), it is obviously suggested that re-expression of miR-22 may have considerable potential for clinical treatment of HCC patients, especially for those with poor miR-22 expression in tumour tissue.

Human HDAC4, an important epigenetic modifier, is further identified to be a direct target of miR-22 in HCC. A current report also revealed that miR-22 can repress Max expression and cell-cycle progression regulated by Myc–Max complex (Ting *et al*, 2010). These results, together with ER*α* above, are consistent with current opinions that a single miRNA can target multiple mRNAs, named ‘targetome’, to post-transcriptionally regulate gene expression (Selbach *et al*, 2008). Hence, it is probable that we are still far from unveiling the last target of miR-22, and perhaps the accumulation of all these targets, including Max and HDAC4, constitutes the phenotype of miR-22 restoration in HCC cells. According to this presumption, interesting future work may be carried out to identify the ‘targetome’ and the entire roles of miR-22 in cancer development.
