# Supplementary material for: microRNA-22, downregulated in hepatocellular carcinoma and correlated with prognosis, suppresses cell proliferation and tumourigenicity
Source: Br J Cancer. 2010 Sep 14;103(8):1215–20. doi: 10.1038/sj.bjc.6605895 (PMC2967065; doi:10.1038/sj.bjc.6605895)
Supplement: Supplementary Table 1 [file 6605895x1.doc]

**Supplementary Table 1. Characteristics of HCC patients used in this study**

| No. | Age  (year) | Sex | HBV | | | | | HCV | | Liver Cirrhosis | HCC | | | |
| --- | --- | --- | --- | --- | --- | --- | --- | --- | --- | --- | --- | --- | --- | --- |
|  |  |  | HB sAg | HB sAb | HB eAg | HB eAb | HB cAb | | HCV Ab |  | Origin | TNM Stage | Histological Grade | AFP  (ng/ml) |
|  |  |  |  |  |  |  |  |  |  |  |  |  |  |  |
| 1 | 38 | M | + | — | — | + | + | | — | + | primary HCC | T2 N0 M0 | Ⅲ | >1210 |
| 2 | 45 | M | + | — | — | + | + | | — | + | primary HCC | T2 N0 M0 | Ⅱ | 339.1 |
| 3 | 43 | M | + | — | — | + | + | | — | + | primary HCC | T2 N0 M0 | Ⅰ | 211.7 |
| 4 | 46 | M | + | — | + | — | + | | — | + | primary HCC | T2 N0 M0 | Ⅲ | >1210 |
| 5 | 56 | M | + | — | — | + | + | | — | + | primary HCC | T2 N0 M0 | Ⅱ | 35.4 |
| 6 | 64 | M | + | — | — | + | + | | — | — | primary HCC | T2 N0 M0 | Ⅱ | >1210 |
| 7 | 55 | M | + | — | + | — | + | | — | + | primary HCC | T2 N0 M0 | Ⅲ | 38.9 |
| 8 | 61 | M | + | — | — | + | + | | — | + | primary HCC | T2 N0 M0 | Ⅲ | >1210 |
| 9 | 53 | M | + | — | — | + | + | | — | — | primary HCC | T2 N0 M0 | Ⅰ | 2.1 |
| 10 | 53 | M | + | — | + | — | + | | — | — | primary HCC | T2 N0 M0 | Ⅲ | >1210 |
| 11 | 37 | M | + | — | — | + | + | | — | + | primary HCC | T2 N0 M0 | Ⅱ | 57 |
| 12 | 53 | M | + | — | — | + | + | | — | + | primary HCC | T1 N0 M0 | Ⅲ | 403.8 |
| 13 | 61 | M | + | — | — | + | + | | — | + | primary HCC | T2 N0 M0 | Ⅲ | >1210 |
| 14 | 79 | M | + | — | — | + | + | | — | + | primary HCC | T3 N0 M0 | Ⅲ | 2.1 |
| 15 | 59 | M | + | — | — | + | + | | — | — | primary HCC | T2 N0 M0 | Ⅲ | 11.1 |
| 16 | 49 | M | + | — | — | + | + | | — | + | primary HCC | T1 N0 M0 | Ⅱ | >1210 |
| 17 | 54 | M | + | — | — | + | + | | — | + | primary HCC | T2 N0 M0 | Ⅱ | 35.2 |
| 18 | 37 | M | + | — | — | + | + | | — | + | primary HCC | T2 N0 M0 | Ⅲ | 8.6 |
| 19 | 36 | M | + | — | — | + | + | | — | + | primary HCC | T2 N0 M0 | Ⅱ | 10.6 |
| 20 | 59 | M | + | — | — | + | + | | — | + | primary HCC | T2 N0 M0 | Ⅲ | 165.1 |
| 21 | 44 | M | + | — | — | + | + | | — | + | primary HCC | T2 N0 M0 | Ⅲ | 9.8 |
| 22 | 36 | M | + | — | — | + | + | | — | + | primary HCC | T2 N0 M0 | Ⅲ | >1210 |
| 23 | 35 | M | + | — | — | + | + | | — | + | primary HCC | T2 N0 M0 | Ⅲ | >1210 |
| 24 | 45 | M | — | + | — | — | + | | — | — | primary HCC | T1 N0 M0 | Ⅲ | 21.4 |
| 25 | 59 | F | + | — | — | + | + | | — | + | primary HCC | T1 N0 M0 | Ⅱ | 3.9 |
| 26 | 69 | M | — | — | — | + | + | | — | — | primary HCC | T3 N0 M0 | Ⅰ | 5 |
| 27 | 49 | M | + | — | — | + | + | | — | + | primary HCC | T2 N0 M0 | Ⅲ | >1210 |
| 28 | 48 | M | + | — | — | + | + | | — | + | primary HCC | T2 N0 M0 | Ⅱ | 51.8 |
| 29 | 53 | M | + | — | — | + | + | | — | + | primary HCC | T2 N0 M0 | Ⅱ | >1210 |
| 30 | 48 | M | + | — | — | + | + | | — | + | primary HCC | T3 N0 M0 | Ⅲ | 8.2 |
| 31 | 55 | M | + | — | — | + | + | | — | + | primary HCC | T1 N0 M0 | Ⅲ | >1210 |
| 32 | 57 | M | + | — | — | + | + | | — | + | primary HCC | T2 N0 M0 | Ⅰ | >1210 |
| 33 | 28 | M | + | — | — | + | + | | — | + | primary HCC | T2 N0 M0 | Ⅲ | 304.2 |
| 34 | 59 | M | + | — | — | + | + | | — | + | primary HCC | T2 N0 M0 | Ⅱ | 260.4 |
| 35 | 60 | M | + | — | + | — | + | | — | — | primary HCC | T3 N0 M0 | Ⅲ | >1210 |
| 36 | 38 | M | + | — | — | + | + | | — | + | primary HCC | T2 N0 M0 | Ⅲ | 455.7 |
| 37 | 59 | M | + | — | — | — | + | | — | — | primary HCC | T2 N0 M0 | Ⅲ | 2.2 |
| 38 | 49 | M | + | — | + | — | + | | — | — | primary HCC | T3 N1 M0 | Ⅲ | 37.8 |
| 39 | 46 | M | + | — | — | + | + | | — | + | primary HCC | T1 N0 M0 | Ⅰ | 1.2 |
| 40 | 69 | M | — | — | — | — | — | | — | — | primary HCC | T3 N1 M0 | Ⅲ | 2 |
| 41 | 59 | M | + | — | + | — | + | | — | — | primary HCC | T1 N0 M0 | Ⅱ | 16.4 |
| 42 | 52 | M | + | — | — | + | + | | — | + | primary HCC | T1 N0 M0 | Ⅰ | >1210 |
| 43 | 51 | M | + | — | — | + | + | | — | + | primary HCC | T1 N0 M0 | Ⅲ | 39.4 |
| 44 | 58 | M | + | — | — | + | + | | — | + | primary HCC | T1 N0 M0 | Ⅱ | 1 |
| 45 | 55 | M | + | — | — | + | + | | — | — | primary HCC | T1 N0 M0 | Ⅱ | 16.4 |
| 46 | 38 | M | + | — | + | — | + | | — | + | primary HCC | T2 N0 M0 | Ⅲ | >1210 |
| 47 | 55 | M | + | — | — | + | + | | — | + | primary HCC | T1 N0 M0 | Ⅲ | >1210 |
| 48 | 50 | M | + | — | — | + | + | | — | — | primary HCC | T1 N0 M0 | Ⅰ | 3.7 |
| 49 | 52 | M | + | — | + | — | + | | — | + | primary HCC | T1 N0 M0 | Ⅲ | 6.4 |
| 50 | 54 | M | — | — | — | — | — | | — | + | primary HCC | T1 N0 M0 | Ⅱ | 3 |
| 51 | 61 | F | — | — | — | — | — | | — | + | primary HCC | T3 N1 M0 | Ⅲ | 4.6 |
| 52 | 63 | M | + | — | — | + | + | | — | + | primary HCC | T3 N1 M0 | Ⅱ | >1210 |
| 53 | 53 | M | + | — | — | + | + | | — | + | primary HCC | T3 N1 M0 | Ⅱ | 33.3 |
| 54 | 47 | M | + | — | — | + | + | | — | + | primary HCC | T1 N0 M0 | Ⅱ | >1210 |
| 55 | 23 | F | + | — | — | + | + | | — | + | primary HCC | T3 N3 M0 | Ⅲ | >1210 |
| 56 | 40 | M | + | — | — | + | + | | — | + | primary HCC | T1 N0 M0 | Ⅱ | 4.9 |
| 57 | 58 | M | + | — | — | + | + | | — | + | primary HCC | T3 N3 M0 | Ⅱ | 6.4 |
| 58 | 51 | M | + | — | + | — | + | | — | — | primary HCC | T2 N0 M0 | Ⅲ | 149.1 |
| 59 | 44 | M | + | — | — | + | + | | — | + | primary HCC | T3 N1 M0 | Ⅲ | 16.7 |
| 60 | 63 | M | + | — | + | — | + | | — | + | primary HCC | T2 N0 M0 | Ⅱ | 5.3 |
| 61 | 58 | M | + | — | + | — | + | | — | — | primary HCC | T3 N0 M0 | Ⅰ | 2.5 |
| 62 | 41 | M | + | — | — | + | + | | — | + | primary HCC | T1 N0 M0 | Ⅱ | 61.6 |
| 63 | 48 | M | + | — | + | — | + | | — | — | primary HCC | T1 N0 M0 | Ⅱ | >1210 |
| 64 | 45 | M | + | — | — | + | + | | — | + | primary HCC | T1 N0 M0 | Ⅲ | 5.5 |
| 65 | 51 | M | + | — | — | + | + | | — | + | primary HCC | T1 N0 M0 | Ⅱ | >1210 |
| 66 | 41 | F | — | — | — | — | — | | — | + | primary HCC | T1 N0 M0 | Ⅱ | >1210 |
| 67 | 70 | M | + | — | — | + | + | | — | + | primary HCC | T1 N0 M0 | Ⅲ | 172.8 |
| 68 | 43 | F | — | — | — | — | — | | — | — | primary HCC | T1 N0 M0 | Ⅱ | 5.9 |
| 69 | 37 | M | + | — | — | + | + | | — | — | primary HCC | T1 N0 M0 | Ⅱ | >1210 |
| 70 | 48 | M | ＋ | — | — | + | + | | — | — | primary HCC | T1 N0 M0 | Ⅱ | 492.6 |
| 71 | 72 | M | + | — | — | + | + | | — | — | primary HCC | T1 N0 M0 | Ⅱ | 13.5 |
| 72 | 61 | M | — | + | — | + | — | | — | + | primary HCC | T1 N0 M0 | Ⅱ | 2.6 |
| 73 | 61 | M | + | — | — | + | + | | — | + | primary HCC | T1 N0 M0 | Ⅱ | >1210 |
| 74 | 54 | F | + | — | — | + | + | | — | — | primary HCC | T1 N0 M0 | Ⅱ | 35.2 |
| 75 | 46 | M | + | — | — | + | + | | — | + | primary HCC | T1 N0 M0 | Ⅲ | >1210 |
| 76 | 52 | M | + | — | — | + | + | | — | + | primary HCC | T1 N0 M0 | Ⅲ | 57.8 |
| 77 | 45 | M | + | — | — | + | + | | — | — | primary HCC | T1 N0 M0 | Ⅰ | 768.4 |
| 78 | 50 | M | + | — | — | + | + | | — | + | primary HCC | T2 N0 M0 | Ⅱ | 6.7 |
| 79 | 43 | M | + | — | — | + | + | | — | + | primary HCC | T1 N0 M0 | Ⅱ | >1210 |
| 80 | 71 | M | + | — | — | + | + | | — | + | primary HCC | T1 N0 M0 | Ⅱ | >1210 |
| 81 | 33 | M | — | — | — | — | — | | — | — | primary HCC | T1 N0 M0 | Ⅱ | 1111.7 |
| 82 | 30 | F | + | — | — | + | + | | — | — | primary HCC | T3 N1 M0 | Ⅱ | 209.3 |
| 83 | 51 | M | + | — | — | + | + | | — | — | primary HCC | T3 N2 M0 | Ⅱ | 9.6 |
| 84 | 52 | M | + | — | + | — | + | | — | + | primary HCC | T1 N0 M0 | Ⅲ | 17.2 |
| 85 | 35 | M | + | — | — | + | + | | — | + | primary HCC | T1 N0 M0 | Ⅱ | >1210 |
| 86 | 45 | M | + | — | — | + | + | | — | + | primary HCC | T1 N0 M0 | Ⅰ | 54.1 |
| 87 | 58 | M | + | — | + | — | + | | — | + | primary HCC | T1 N0 M0 | Ⅰ | 10.5 |
| 88 | 47 | M | + | — | + | — | + | | — | + | primary HCC | T1 N0 M0 | Ⅱ | >1210 |
| 89 | 44 | M | + | — | — | + | + | | — | + | primary HCC | T3 N2 M0 | Ⅰ | 261.70 |
| 90 | 49 | M | + | — | — | + | + | | — | + | primary HCC | T3 N1 M0 | Ⅲ | 9.4 |
| 91 | 61 | M | + | — | — | + | + | | — | + | primary HCC | T1 N0 M0 | Ⅱ | 28.6 |
| 92 | 43 | M | + | — | — | — | — | | — | + | primary HCC | T3 N1 M0 | Ⅲ | >1210 |
| 93 | 52 | M | + | — | — | + | + | | — | — | primary HCC | T3 N1 M0 | Ⅲ | >1210 |
| 94 | 69 | F | + | — | + | — | + | | — | + | primary HCC | T3 N3 M0 | Ⅱ | 10.00 |
| 95 | 45 | M | + | — | — | + | + | | — | + | primary HCC | T2 N0 M0 | Ⅱ | 388.4 |
| 96 | 30 | M | — | — | — | — | — | | + | + | primary HCC | T3 N2 M0 | Ⅳ | 59.8 |
| 97 | 46 | M | + | — | — | + | + | | — | + | primary HCC | T1 N0 M0 | Ⅲ | >1210 |
| 98 | 41 | M | + | — | — | + | + | | — | + | primary HCC | T1 N0 M0 | Ⅲ | >1210 |
| 99 | 38 | M | + | — | — | + | + | | — | + | primary HCC | T3 N1 M0 | Ⅲ | 21.2 |
| 100 | 55 | M | + | — | — | + | + | | — | + | primary HCC | T3 N1 M0 | Ⅱ | 134 |
| 101 | 52 | F | + | — | — | + | + | | — | + | primary HCC | T1 N0 M0 | Ⅲ | >1210 |
| 102 | 47 | M | + | — | — | + | + | | — | + | primary HCC | T1 N0 M0 | Ⅲ | >1210 |
| 103 | 60 | M | + | — | — | + | + | | — | + | primary HCC | T3 N2 M0 | Ⅲ | >1210 |
| 104 | 42 | M | + | — | — | + | + | | — | + | primary HCC | T3 N1 M0 | Ⅲ | >1210 |
| 105 | 44 | M | + | — | — | + | + | | — | + | primary HCC | T1 N0 M0 | Ⅲ | >1210 |
| 106 | 47 | M | + | — | — | + | + | | — | + | primary HCC | T1 N0 M0 | Ⅲ | 3.80 |
| 107 | 38 | F | — | — | — | — | — | | — | + | primary HCC | T1 N0 M0 | Ⅲ | >1210 |
| 108 | 45 | F | + | — | — | + | + | | — | — | primary HCC | T3 N1 M0 | Ⅲ | 339.1 |
| 109 | 43 | M | + | — | — | + | + | | — | + | primary HCC | T2 N0 M0 | Ⅲ | 211.7 |
| 110 | 51 | M | — | — | — | — | — | | — | + | primary HCC | T3 N1 M0 | Ⅲ | >1210 |
| 111 | 42 | M | + | — | — | + | + | | — | + | primary HCC | T1 N0 M0 | Ⅱ | 35.4 |
| 112 | 71 | F | + | — | — | + | + | | — | + | primary HCC | T1 N0 M0 | Ⅱ | >1210 |
| 113 | 55 | M | + | — | — | + | + | | — | + | primary HCC | T1 N0 M0 | Ⅱ | 38.9 |
| 114 | 48 | M | + | — | — | + | + | | — | + | primary HCC | T1 N0 M0 | Ⅲ | >1210 |
| 115 | 50 | M | + | — | — | + | + | | — | + | primary HCC | T3 N1 M0 | Ⅲ | 2.1 |
| 116 | 41 | M | + | — | — | + | + | | — | + | primary HCC | T3 N2 M0 | Ⅲ | >1210 |
| 117 | 65 | M | + | + | — | + | — | | — | + | primary HCC | T1 N0 M0 | Ⅲ | 57 |
| 118 | 52 | M | + | — | — | + | + | | — | + | primary HCC | T1 N0 M0 | Ⅱ | 403.8 |
| 119 | 35 | M | + | — | — | + | + | | — | + | primary HCC | T1 N0 M0 | Ⅰ | >1210 |
| 120 | 55 | M | + | — | — | + | + | | — | + | primary HCC | T2 N0 M0 | Ⅲ | 2.1 |
| 121 | 20 | M | + | — | — | + | + | | — | — | primary HCC | T3 N3 M0 | Ⅲ | 11.1 |
| 122 | 48 | M | + | — | — | + | + | | — | + | primary HCC | T2 N0 M0 | Ⅲ | >1210 |
| 123 | 40 | M | + | — | — | + | + | | — | + | primary HCC | T2 N0 M0 | Ⅱ | 35.2 |
| 124 | 58 | M | — | — | — | + | + | | — | + | primary HCC | T3 N3 M0 | Ⅱ | 8.6 |
| 125 | 59 | M | + | — | + | + | — | | — | + | primary HCC | T1 N0 M0 | Ⅱ | 10.6 |
| 126 | 31 | F | + | — | — | + | + | | — | — | primary HCC | T1 N0 M0 | Ⅱ | 165.1 |
| 127 | 31 | M | + | — | — | + | + | | — | + | primary HCC | T1 N0 M0 | Ⅲ | 9.8 |
| 128 | 70 | M | + | — | — | + | + | | — | + | primary HCC | T1 N0 M0 | Ⅱ | >1210 |
| 129 | 45 | M | + | — | — | + | + | | — | + | primary HCC | T3 N1 M0 | Ⅱ | >1210 |
| 130 | 60 | F | + | — | — | + | + | | — | + | primary HCC | T1 N0 M0 | Ⅲ | 21.4 |
| 131 | 60 | M | + | — | — | + | + | | — | + | primary HCC | T3 N1 M0 | Ⅱ | 3.9 |
| 132 | 54 | M | + | — | — | + | + | | — | + | primary HCC | T3 N1 M0 | Ⅱ | 5 |
| 133 | 60 | M | + | — | — | + | + | | — | + | primary HCC | T1 N0 M0 | Ⅲ | >1210 |
| 134 | 45 | M | + | — | — | + | + | | — | + | primary HCC | T3 N2 M0 | Ⅱ | 51.8 |
| 135 | 52 | M | — | + | — | — | + | | — | + | primary HCC | T3 N1 M0 | Ⅲ | >1210 |
| 136 | 46 | M | + | — | — | + | + | | — | + | primary HCC | T1 N0 M0 | Ⅰ | 8.2 |
| 137 | 53 | M | + | — | — | + | + | | — | — | primary HCC | T1 N0 M0 | Ⅱ | >1210 |
| 138 | 60 | M | — | + | — | — | + | | — | + | primary HCC | T4 N0 M0 | Ⅱ | >1210 |
| 139 | 58 | F | + | — | — | + | + | | — | — | primary HCC | T3 N1 M0 | Ⅱ | 304.2 |
| 140 | 40 | M | + | — | — | + | + | | — | + | primary HCC | T3 N1 M0 | Ⅲ | 260.4 |
| 141 | 40 | M | + | — | — | + | + | | — | — | primary HCC | T4 N0 M0 | Ⅱ | >1210 |
| 142 | 70 | M | + | + | — | + | — | | — | + | primary HCC | T1 N0 M0 | Ⅲ | 455.7 |
| 143 | 32 | M | + | — | — | + | + | | — | + | primary HCC | T3 N1 M0 | Ⅲ | 2.2 |
| 144 | 45 | M | + | — | — | + | + | | — | + | primary HCC | T3 N1 M0 | Ⅱ | 37.8 |
| 145 | 51 | M | + | — | — | + | + | | — | + | primary HCC | T1 N0 M0 | Ⅲ | 1.2 |
| 146 | 42 | F | + | — | — | + | + | | — | — | primary HCC | T1 N0 M0 | Ⅲ | 2 |
| 147 | 71 | M | + | — | — | + | + | | — | + | primary HCC | T3 N2 M0 | Ⅲ | 16.4 |
| 148 | 55 | M | + | — | — | + | + | | — | + | primary HCC | T3 N1 M0 | Ⅲ | >1210 |
| 149 | 48 | M | — | — | — | + | + | | — | + | primary HCC | T1 N0 M0 | Ⅲ | 39.4 |
| 150 | 50 | M | + | — | + | + | — | | — | + | primary HCC | T1 N0 M0 | Ⅲ | 1 |
| 151 | 41 | M | + | — | — | + | + | | — | — | primary HCC | T1 N0 M0 | Ⅲ | 16.4 |
| 152 | 65 | F | + | — | — | + | + | | — | + | primary HCC | T3 N1 M0 | Ⅲ | >1210 |
| 153 | 52 | F | + | — | — | + | + | | — | + | primary HCC | T2 N0 M0 | Ⅲ | >1210 |
| 154 | 35 | M | + | — | — | + | + | | — | + | primary HCC | T3 N1 M0 | Ⅲ | 3.7 |
| 155 | 55 | M | + | — | — | + | + | | — | + | primary HCC | T1 N0 M0 | Ⅱ | 6.4 |
| 156 | 20 | M | + | — | — | + | + | | — | + | primary HCC | T1 N0 M0 | Ⅱ | 3 |
| 157 | 48 | F | + | — | — | + | + | | — | + | primary HCC | T1 N0 M0 | Ⅱ | 4.6 |
| 158 | 40 | M | + | — | — | + | + | | — | + | primary HCC | T1 N0 M0 | Ⅲ | >1210 |
| 159 | 58 | M | + | — | — | + | + | | — | + | primary HCC | T3 N1 M0 | Ⅲ | 33.3 |
| 160 | 59 | M | + | — | — | + | + | | — | + | primary HCC | T3 N2 M0 | Ⅲ | >1210 |

Abbreviations: HBV, hepatitis B virus; HCV, hepatitis C virus; HCC, hepatocellular carcinoma; TNM, tumor-node-metastasis; AFP, alpha-fetoprotein.
